# The Effects of Ibogaine on Uterine Smooth Muscle Contractions: Relation to the Activity of Antioxidant Enzymes

**DOI:** 10.1155/2018/5969486

**Published:** 2018-02-11

**Authors:** Zorana Oreščanin-Dušić, Nikola Tatalović, Teodora Vidonja-Uzelac, Jelena Nestorov, Aleksandra Nikolić-Kokić, Ana Mijušković, Mihajlo Spasić, Roman Paškulin, Mara Bresjanac, Duško Blagojević

**Affiliations:** ^1^Department of Physiology, Institute for Biological Research “Siniša Stanković”, University of Belgrade, Despota Stefana 142, 11000 Belgrade, Serbia; ^2^Department of Biochemistry, Institute for Biological Research “Siniša Stanković”, University of Belgrade, Despota Stefana 142, 11000 Belgrade, Serbia; ^3^OMI Institute, Trnovska 8, SI-1000 Ljubljana, Slovenia; ^4^Institute of Pathophysiology, University of Ljubljana, Vrazov trg 2, SI-1000 Ljubljana, Slovenia

## Abstract

Ibogaine is an indole alkaloid originally extracted from the root bark of the African rainforest shrub *Tabernanthe iboga*. It has been explored as a treatment for substance abuse because it interrupts drug addiction and relieves withdrawal symptoms. However, it has been shown that ibogaine treatment leads to a sharp and transient fall in cellular ATP level followed by an increase of cellular respiration and ROS production. Since contractile tissues are sensitive to changes in the levels of ATP and ROS, here we investigated an ibogaine-mediated link between altered redox homeostasis and uterine contractile activity. We found that low concentrations of ibogaine stimulated contractile activity in spontaneously active uteri, but incremental increase of doses inhibited it. Inhibitory concentrations of ibogaine led to decreased SOD1 and elevated GSH-Px activity, but doses that completely inhibited contractions increased CAT activity. Western blot analyses showed that changes in enzyme activities were not due to elevated enzyme protein concentrations but posttranslational modifications. Changes in antioxidant enzyme activities point to a vast concentration-dependent increase in H_2_O_2_ level. Knowing that extracellular ATP stimulates isolated uterus contractility, while H_2_O_2_ has an inhibitory effect, this concentration-dependent stimulation/inhibition could be linked to ibogaine-related alterations in ATP level and redox homeostasis.

## 1. Introduction

Ibogaine is a psychoactive indole alkaloid derived from the rainforest shrub *Tabernanthe iboga*, which grows in West Africa. The tribes of Gabon have used the iboga plant root bark as a stimulant, for medicinal purposes, and in rite of passage ceremonies, for centuries. The pharmacological properties of ibogaine have been known for over 100 years. During the early period of exploration, ibogaine was mostly known for its ability to inspire a sense of wellbeing both mentally and physically. Ibogaine has also been used for the treatment of substance abuse because it interrupts drug addiction, relieves withdrawal symptoms, and significantly decreases the desire for cocaine, heroin, alcohol, and most other mind-altering drugs [1–4]. The pharmacology of ibogaine is quite complex and affects many different neurotransmitter systems simultaneously. Ibogaine binds to several types of receptors: 5-hydroxytryptamine (5-HT), opioid, nicotinic, and *N*-methyl-d-aspartate (NMDA), as well as dopaminergic and 5-HT transporters and monoamine oxidase (MAO) enzyme [5–7].

Paskulin et al. have shown that ibogaine causes a sharp and transient fall in the cellular ATP level in yeast, which was followed by an immediate increase in respiration and CO_2_ production, in a time- and concentration-dependent manner [8, 9]. Increased respiration leads to an increase in ROS production, changes in cellular redox state, and subsequent activation of antioxidant enzymes. These effects of ibogaine are not mediated by receptor binding [8, 10]. The proteome changes (induction of energy metabolism enzymes, antioxidant enzymes, and numerous low-abundance proteins) are responsible for at least a part of the initial energy expenditure in ibogaine-treated yeast [8, 11]. A study on human blood erythrocytes also showed that ibogaine leads to the release of ATP and the elevation of antioxidant activity [10]. Ibogaine does not have any significant *in vitro* antioxidant properties per se, so its influence on the physiological oxidative stress defense system has been suggested to be in a proantioxidant manner [9, 10].

Contractile tissues are sensitive to ATP levels, and the depletion of energetics could lead to the impairment of regular rhythms and reversible inhibition of contractile activity [12]. Extracellular ATP is known to stimulate uterine contractions in different species, but the exact underlying mechanisms are poorly investigated [13]. Furthermore, different contractile tissues, including uterus, are also sensitive to changes of the cellular redox state and ROS [14], especially hydrogen peroxide (H_2_O_2_) [15, 16]. Moreover, the antioxidant enzyme cytosolic copper-zinc-containing superoxide dismutase (SOD1) can also affect the contractility of uterine smooth muscles [17]. These points out that cellular energetic consumption, subsequent redox modifications, and antioxidant defense make an intracellular net that influences smooth muscle contractility.

Keeping in mind the effects of ibogaine on cellular energy and ROS metabolism, the aim of this study was to investigate the effects of ibogaine on the activity and expression of antioxidant enzymes in isolated rat uteri and a possible link between ibogaine-altered redox homeostasis, antioxidant defense, and the contractile properties of the uterus. To that end, we studied the effect of increasing concentrations of ibogaine (1, 2, 5, 10, 15, 20, 40, and 60 mg/l) on an isolated rat uterus in conditions of low (spontaneous) and high (Ca^2+^-stimulated) intensity of contractions, as well as the expression and activity of antioxidant enzymes: cytosolic copper-zinc-containing superoxide dismutase (SOD1), mitochondrial manganese-containing superoxide dismutase (SOD2), catalase (CAT), glutathione peroxidase (GSH-Px), and glutathione reductase (GR).

## 2. Materials and Methods

### 2.1. Materials

The ibogaine hydrochloride employed in the study came from two sources: one source (PubChem CID: 197059), purity 98.93%, was donated by Sacrament of Transition, Maribor, Slovenia. The other source was Remøgen (Phytostan Enterprises Inc., Canada), 99.9% pure ibogaine HCl, provided by MB and PI of the LNPR project under ARRS Programme P3-0171.

### 2.2. Animals

The procedures complied with the EEC Directive on the protection of animals used for experimental and other scientific purposes and were approved by the Ethical Committee for the Use of Laboratory Animals of the Institute for the Biological Research “Siniša Stanković,” University of Belgrade. Animals were kept at 22°C, housed 3 per cage, and fed ad libitum. Experiments were performed on isolated uteri from female virgin Wistar rats three months old (200–250 g) in the estrus phase of the estrous cycle, which was determined by examination of a daily vaginal lavage [18].

### 2.3. Isolated Organ Bath Studies

All rats were sacrificed by rapid decapitation. The uterine horns were rapidly excised and carefully cleaned of surrounding connective tissues and mounted vertically in a 10 ml volume organ bath containing De Jalon's solution (in g/l: NaCl 9.0, KCl 0.42, NaHCO_3_ 0.5, CaCl_2_ 0.06, and glucose 0.5), under initial tension of 1 g, aerated with a mixture of 95% oxygen and 5% carbon dioxide at 37°C. After an equilibration period (about 30 min), uteri achieved stable spontaneous contractions (this activity is referred to as spontaneous contractions). Another set of uteri was additionally activated by the addition of 0.66 g/l of CaCl_2_ in an organ bath (referred to as Ca^2+^-stimulated contractions) to achieve maximal intensity of phasic contractions. The effect of ibogaine on uterine contractility was studied by cumulative ibogaine addition up to complete contractility inhibition (concentrations: 1, 2, 5, 10, 15, 20, and 40 mg/l for spontaneously active uteri and 1, 2, 5, 10, 15, 20, 40, and 60 mg/l for Ca^2+^-stimulated uteri). Myometrial tension was recorded isometrically with a TSZ-04-E isolated organ bath and transducer (MDE Research, Heidelberg, Germany). To determine if ibogaine effects were reversible, uteri were washed out after treatment by De Jalon's solution. For corresponding controls, both untreated spontaneously active and Ca^2+^-stimulated uteri placed in an isolated organ bath parallel to treated samples were used.

In order to determine the effect of ibogaine inhibitory concentrations on antioxidant enzyme activities in uteri, an additional group of both spontaneously active and Ca^2+^-stimulated active uteri were treated with increasing concentrations of ibogaine up to 20 mg/l, which was shown as the cumulative dose that inhibited uterine contractions up to about 50% of controls.

After the described treatments, samples were immediately frozen using liquid nitrogen and transferred to −80°C until further analysis.

### 2.4. Determination of Antioxidant Enzyme Activities

Thawed uteri were homogenized and sonicated in 0.25 M sucrose, 1 mM ethylenediaminetetraacetic acid, and 0.05 M Tris-HCl buffer (pH 7.4) before centrifugation for 90 min at 105,000 *×*g. The supernatant was used to determine enzyme activities. Total superoxide dismutase (SOD) activity was determined by the adrenaline method [19]. One SOD unit was defined as the amount of the enzyme necessary to decrease the rate of adrenalin auto-oxidation by 50% at pH 10.2. For determination of SOD2 activity, the assay was performed after preincubation with 8 mM KCN. The SOD1 activity was calculated as the difference between the total SOD and SOD2 activities. CAT activity was determined by the monitoring of hydrogen peroxide consumption [20] and expressed in U/mg protein. The activity of GSH-Px was determined using *t*-butyl hydroperoxide as a substrate [21] and expressed in mU/mg protein. GR activity was determined by NADPH oxidation concomitant with GSSG reduction [22] and expressed in mU/mg protein. Protein concentration was measured by the method of Lowry et al. [23]. All measurements of absorbance were performed using a Shimadzu UV-160 spectrophotometer (Shimadzu Scientific Instruments, Shimadzu Corporation, Kyoto, Japan).

### 2.5. SDS-PAGE and Immunoblotting

Whole cell extracts from control and treated uteri were mixed 1 : 1 with Laemmli sample buffer, boiled for 5 min, and stored at −80°C until further analysis. Proteins were resolved in 12% SDS polyacrylamide gels and transferred to PVDF membranes. SOD1, SOD2, CAT, GSH-Px, and GR were detected by Abcam antibodies (ab13498, ab13533, ab16731, ab22604, and ab16801, resp.). *β*-Actin was detected by AC-15 antibody (Sigma-Aldrich). After incubation with secondary antibodies, the immunoreactive proteins were visualized by the enhanced chemifluorescence (ECF) method (Amersham Biosciences Limited, UK). Quantitative analysis of immunoreactive bands was performed using the ImageQuant software. *β*-Actin was used as equal load control.

### 2.6. Data Analysis and Statistical Procedures

Statistical analyses were performed according to the protocols described by Hinkle et al. [24]. The effects of treatments on uterine contractions were calculated as percentages of control. Contractions were analyzed for the force of contraction, amplitude, and frequency. Each data value is expressed as mean ± SEM. The forces of contractions of spontaneously active uteri and Ca^2+^-stimulated uteri during the control period was compared using a *t*-test (significance: *p* < 0.05). The effect of ibogaine on uterine contractility was tested by two-way ANOVA (factors: type of contractions and concentration of ibogaine) on logarithmically transformed data and post hoc compared by Tukey's HSD test (significance: *p* < 0.05). Sigmoid concentration-response curves for ibogaine treatment were fitted according to Boltzmann functions (the concentration axis was linear), and the concentration required for the half-maximal effect (EC_50_) was calculated. Fitted curves were compared using an *F*-test. EC_50_ values were compared using the *t*-test (significance: *p* < 0.05). The activities and protein expression levels of antioxidant enzymes were compared by one-way ANOVA followed by Tukey's HSD post hoc test (significance: *p* < 0.05). Differences in the activity of antioxidant enzymes in spontaneously active versus Ca^2+^-stimulated uteri were compared using the *t*-test (significance: *p* < 0.05).

## 3. Results

### 3.1. Effects of Increasing Concentrations of Ibogaine on Uterine Contractions

**Figure 1:**
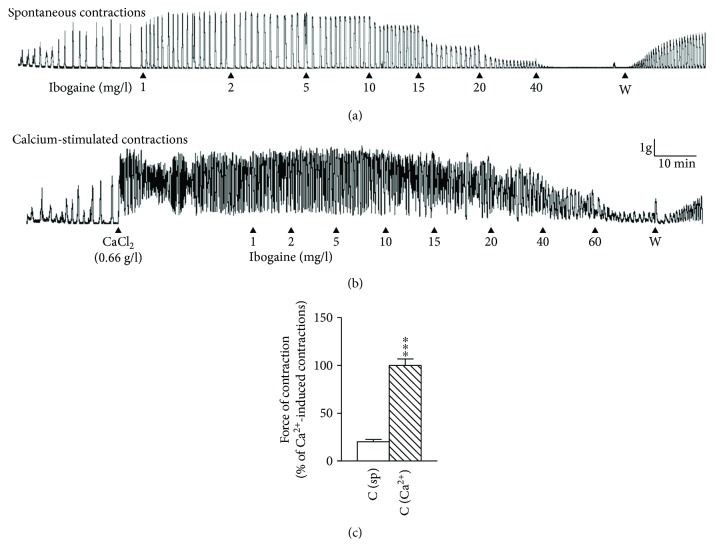
The effects of increasing concentrations of ibogaine on uterine contractions. (a) Representative original trace of spontaneously active uterus treated with increasing concentrations of ibogaine (1–40 mg/l); W = washing out. (b) Representative original trace of Ca^2+^-stimulated uterus treated with increasing concentrations of ibogaine (1–60 mg/l). (c) Force of contractions of spontaneously active and Ca^2+^-stimulated active uteri during the control period, before the addition of ibogaine, calculated as area under the curve per minute. Values are means ± SEM (*n* = 7) and presented as percentage of Ca^2+^-stimulated contractions. Difference was tested by the *t*-test. ^∗∗∗^*p* < 0.001.

**Figure 2:**
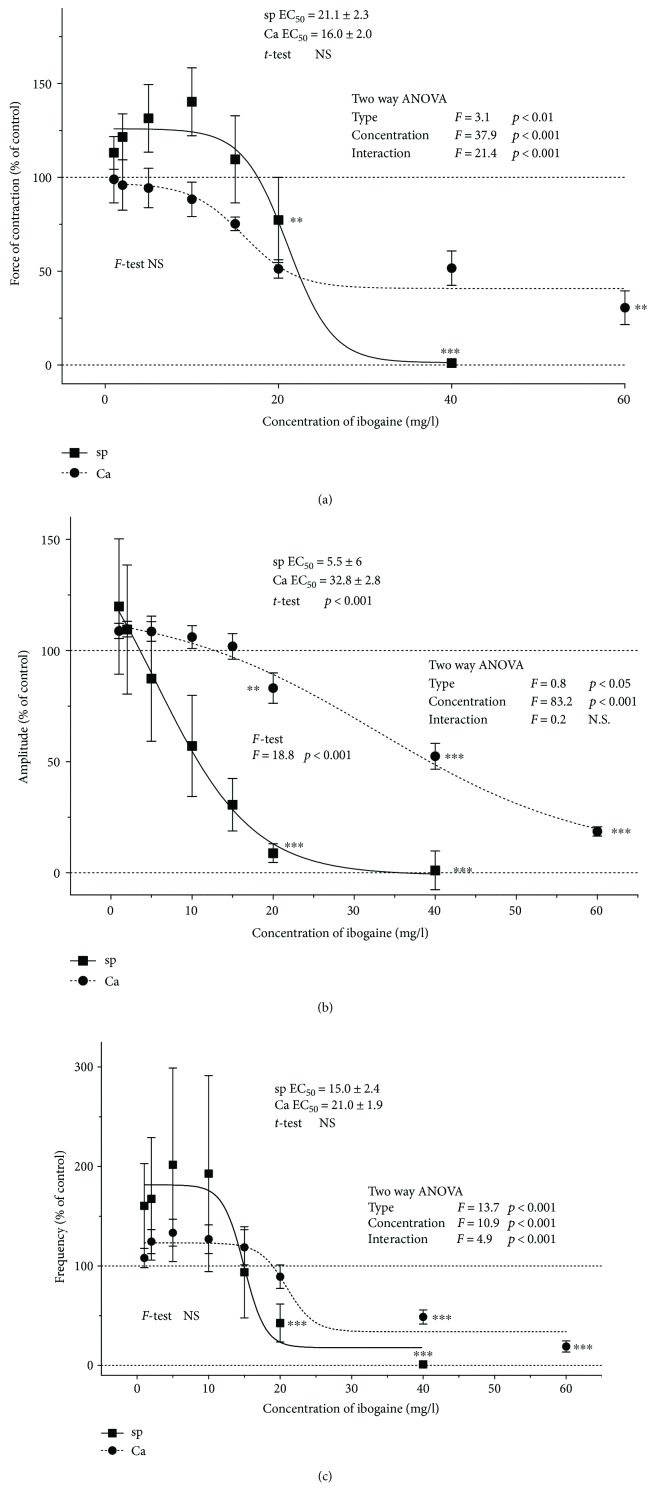
Concentration-response sigmoid fit curves for ibogaine-treated uteri: (a) force of contractions, (b) amplitude, and (c) frequency. Force of contractions, amplitudes, and frequencies were calculated as percentage of controls and expressed as mean values ± SEM (*n* = 7). Differences were tested by two-way ANOVA (factors: type of contraction and concentration of ibogaine). The sigmoid fits were performed according to the Boltzmann equation (fits were compared by the *F*-test) and EC_50_ values were expressed. Differences between EC_50_ values were tested by the *t*-test. ^∗∗^*p* < 0.01, ^∗∗∗^*p* < 0.001.

Ibogaine in concentrations applied in our experiment significantly influenced the contractile activity of uterus in a dose-dependent manner, but effects on spontaneously active and Ca^2+^-stimulated active rat uteri were different and depended on the applied concentration (Figures 1 and 2; ANOVA effects of both concentration, *p* < 0.001, and interactions, *p* < 0.001). In spontaneously active uteri, low concentrations increased the force of contraction and frequency, while higher concentrations caused a dose-dependent decrease in complete contractile activity (Figures 1 and 2; significant ANOVA concentration effect and sigmoidal fit for the observed data). Cumulative concentrations of 40 mg/l led to the complete inhibition of the contractile activity of the spontaneously active rat uterus (Figures 1(a) and 2). On the other hand, ibogaine had no stimulating effect on the force of contraction in Ca^2+^-stimulated uteri, although a small increase (but statistically not significant) in amplitude and frequency is noted at low concentrations (Figures 1(b) and 2). Higher concentrations of ibogaine caused a dose-dependent decrease in contractile activity. A cumulative concentration of 60 mg/l has led to an almost complete inhibition of contractile activity (Figures 1(b) and 2). The force of contractions of Ca^2+^-stimulated uteri was about 5 times greater than that in spontaneously active uteri (Figure 1(c)), and the concentration of ibogaine effective for the inhibition of spontaneously active uteri is lower than that for Ca^2+^-stimulated uteri (significant difference ANOVA for type of contraction effect for all three calculated parameters, as well as for calculated EC_50_ concentrations (*t*-test, *p* < 0.001) and curve shape for amplitude obtained by the *F*-test (*p* < 0.001), Figure 2).

The cease of contractility was reversible in both spontaneously active and Ca^2+^-stimulated rat uteri, since washing out of isolated ibogaine-treated uteri restored their contractile activity (Figures 1(a) and 1(b)).

### 3.2. Effects of Increasing Concentrations of Ibogaine on the Activity of Antioxidant Enzymes

**Figure 3:**
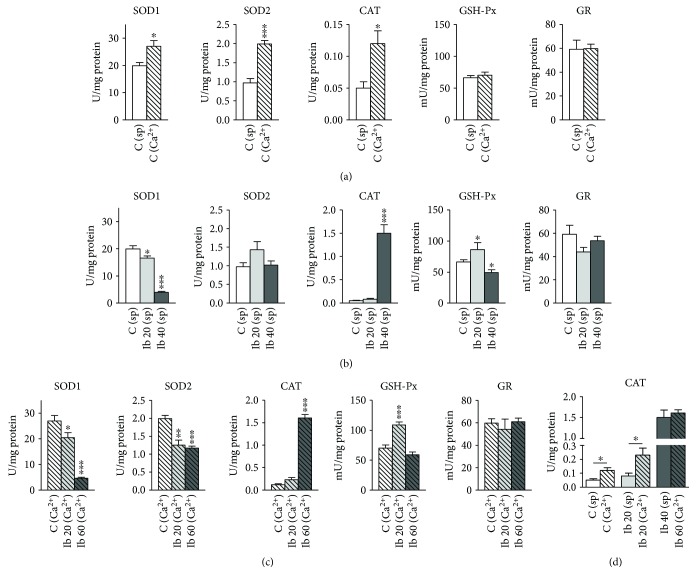
The activity of antioxidant enzymes in the uterus after treatment with increasing concentrations of ibogaine. (a) The activity of antioxidant enzymes in spontaneously active and Ca^2+^-stimulated active uteri. (b) Spontaneously active uteri treated with increasing concentrations of ibogaine (up to 20 and 40 mg/l). (c) Ca^2+^-stimulated active uteri treated with increasing concentrations of ibogaine (up to 20 and 60 mg/l). (d) The activity of CAT in both spontaneously active and Ca^2+^-stimulated active uteri. Data are expressed as mean ± SEM (*n* = 7). For (a) and (d), statistical significance was tested using the *t*-test. For (b) and (c), statistical significance was tested by one-way ANOVA and post hoc compared by Tukey's HSD test. ^∗∗∗^*p* < 0.001, ^∗∗^*p* < 0.01, and ^∗^*p* < 0.05.

In nontreated active control Ca^2+^-stimulated uteri, SOD1, SOD2, and CAT activities were 30%, 2-fold, and 2.4-fold higher, respectively, compared to spontaneously active uteri for the same period of contractile activity (Figure 3(a)). At the same time, GSH-Px and GR activities were at the same level.

In both spontaneously active and Ca^2+^-stimulated active uteri, cumulative concentrations of ibogaine up to 20 mg/l (which caused an inhibition of about 50% of contractile activity) decreased the SOD1 activity and increased the GSH-Px activity compared to corresponding controls (Figures 3(b) and 3(c)). Further addition of ibogaine up to a total cease in contractile activity (40 mg/l and 60 mg/l for spontaneously active and Ca^2+^-stimulated active uteri, resp.) led to a further decrease in SOD1 activity to less than 20% of its initial activity, a multiple increase in CAT activity (a 30-fold and a 13-fold increase compared to the control in spontaneously active and Ca^2+^-stimulated active uteri, resp.), and a fall in previously elevated GSH-Px (Figures 3(b) and 3(c)). The levels of CAT activity after ibogaine treatment up to the inhibition of contractile activity were about the same in both spontaneously active and Ca^2+^-stimulated active uteri (Figure 3(d)). Besides, ibogaine treatment of Ca^2+^-stimulated uteri decreased the SOD2 activity at both experimental points of 20 mg/l and 60 mg/l. There were no significant changes in SOD2 activity in spontaneously active uteri during ibogaine treatment.

### 3.3. Effects of Increasing Concentrations of Ibogaine on the Protein Expression Levels of Antioxidant Enzymes

**Figure 4:**
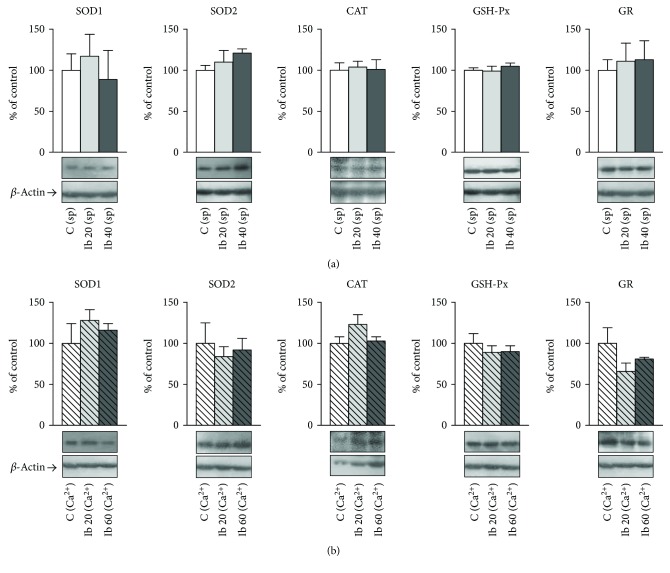
Protein levels of antioxidant enzymes in the uterus after treatment with increasing concentrations of ibogaine. (a) Protein levels of antioxidant enzymes in spontaneously active uteri (representative Western blots are shown underneath the corresponding charts). (b) Protein levels of antioxidant enzymes in Ca^2+^-stimulated uteri (representative Western blots are shown underneath the corresponding charts). Whole cell extracts from uteri (50 *μ*g protein) were subjected to SDS-PAGE and Western blotting. *β*-Actin was used as loading control. Representative Western blots and relative quantification of antioxidant enzyme levels of control uteri (C). Uteri treated with increasing concentrations of ibogaine up to both 20 mg/l and 40 mg/l are shown. Values are means ± SEM (*n* = 6) and are presented as percentage of control.

The protein expression levels of antioxidant enzymes in response to ibogaine treatment remained unaltered (Figure 4). Both spontaneously active and Ca^2+^-stimulated uteri, at both experimental points (20 mg/l and 40 mg/l for spontaneously active and 20 mg/l and 60 mg/l of ibogaine for Ca^2+^-stimulated uteri), showed no significant changes comparing to controls.

## 4. Discussion

Despite its pharmacological properties, it was shown that ibogaine disturbed the redox homeostasis: it provoked ATP depletion and subsequent resynthesis followed by high ROS generation. ATP depletion is rapid after ibogaine addition, and elevated ROS concentration persisted during the following hours [8, 9]. Since it was shown that contractile activity is associated with changes in ROS homeostasis and antioxidant defense, here we studied the physiological response on the level of antioxidant activity to ibogaine application and possible link to uterine contractility.

Our results showed that ibogaine cumulative treatment led to changes in uterine antioxidant activity in parallel to the inhibition of contractile activity. The effects are different for spontaneously active and Ca^2+^-stimulated uteri. Low doses of ibogaine significantly elevated the frequency and contractile activity of already spontaneously active uteri. This effect could be partly attributed to a possible increase in the extracellular concentration of ATP. This assumption is based on the fact that ibogaine liberates ATP from the cellular ATP pool. Our previous results showed that ibogaine releases ATP from human erythrocytes [10]. It is also known that ATP, under the influence of various stimuli, can be released from various cell types through specific transport mechanisms [25, 26] and that addition of extracellular ATP stimulates the contractile activity of the isolated uterus [13]. On the other hand, stimulation of uterine activity by Ca^2+^ led to a significant increase in contractility, to the point that elevation of ATP by ibogaine addition seems insufficient to change the contractility for that type of contraction.

Given that ATP (re)synthesis entails an immediate elevation of ROS, we measured the activity of antioxidant enzymes in uteri at two experimental points: at cumulative concentrations of ibogaine that inhibited uterine contractions up to about 50% of controls (20 mg/l of ibogaine for both types of contractions) and at the end of treatment after inhibition of contractility was obtained (40 mg/l of ibogaine for spontaneously active and 60 mg/l for Ca^2+^-stimulated uteri). In both types of contractile activity, the concentration of 20 mg/l of ibogaine caused an elevation in GSH-Px activity and a decrease in SOD1 activity. On the other hand, the concentration of ibogaine that caused an inhibition of contractile activity led to a 30-fold increase in CAT activity, while SOD1 activity was still decreased. Since the substrate for both GSH-Px and CAT is H_2_O_2_, results indicate high level of hydrogen peroxide after ibogaine addition. Since CAT operates at higher concentration of H_2_O_2_ than GSH-Px, it seems that the production of H_2_O_2_ was higher at higher concentrations of the applied ibogaine. Furthermore, ibogaine decreased the cytosol SOD1 activity in active uteri in a concentration-dependent manner, down to 20% of the initial activity. This further points to the presence of high concentrations of H_2_O_2_ and a gradual increase in H_2_O_2_ in the uterus, since it is known that H_2_O_2_, as a product of SOD, can inhibit its activity by a negative feedback effect [27].

Our previous results [15, 16] showed that H_2_O_2_ can induce concentration-dependent contractile inhibition of isolated rat uteri, mainly via changes in voltage-dependent potassium channels. According to these results, high ibogaine concentration-mediated inhibition of contraction found in the present study may be attributable to the influence of H_2_O_2_ on contractile activity. Inhibition of uterine contractility is transient and reversible: washing ibogaine out of the isolated uterus restores contractile activity, which emphasizes the role of receptor-mediated mechanisms in ibogaine action. Therefore, the role of hydrogen peroxide can be considered as mediatory. However, some other possible mechanisms of ibogaine-induced smooth muscle inhibition of contractility cannot be eliminated, considering its wide range of interaction with different receptors and signal transduction pathways.

An increase in the intensity of smooth muscle contractions induces elevated oxidative metabolism and leads to an increased production of ROS in the system [28]. To investigate whether this is a mechanism by which ibogaine operates at the level of antioxidant activity in spontaneously active uteri or it is the consequence of the direct impact on the energy metabolism, we used Ca^2+^-stimulated uteri. Placing uteri in isotonic solution containing 11-fold higher concentrations of Ca^2+^, we stimulated the influx of Ca^2+^ in uterine muscles and induced a high elevation of contractile activity. In these conditions, uterine contractions as well as energy demands are very intensive (force of contractions 5 times greater compared to spontaneous activity). However, in these conditions, ibogaine showed no initial stimulating effect and caused a further concentration-dependent inhibition of Ca^2+^-stimulated uterine contractions. We found that these conditions stimulate the antioxidant activity by increasing SOD1, SOD2, and CAT activities (30%, 2-fold, and 2.4-fold higher, resp., compared to spontaneous activity) in a similar direction as measured in spontaneously active uteri (the elevation of GSH-Px activity in lower doses and the increase in CAT activity up to the same level as the maximal concentration of ibogaine applied to spontaneously active uteri). These suggest that the metabolic effects of ibogaine at ROS and antioxidant enzyme levels are similar in both types of uterine activity, but different in intensity. In Ca^2+^-stimulated uteri, the inhibition of mitochondrial SOD2 activity was also shown. Since mitochondrial SOD2 activity can be also inhibited by H_2_O_2_ [29, 30], a parallel inhibition of both SOD1 and SOD2 in Ca^2+^-stimulated uteri suggests a higher rate of H_2_O_2_. Moreover, levels of CAT after ibogaine treatment reached the same level in both types of uterine activity, suggesting that the existing amount of CAT has reached its highest rate of activity. A large increase in the ATP turnover rate, which is reflected by a concentration-dependent increase in H_2_O_2_ in the system, could only partially be related to increased energy demands caused by stronger contractile activity. Even when the uterus is contracting with maximal intensity in our experiment, the addition of ibogaine leads to additional multiple increases in CAT activity and a decrease in SOD activity, indicating the existence of other ways of ROS/redox disequilibrium induced by ibogaine.

Since there were no changes in the protein levels of any studied antioxidant enzyme (unaltered protein expression) in our experiment, stimulatory/inhibitory changes of its activities found in this work were based on the regulation of existing quantities of enzymes and can be attributed to the intrinsic characteristics of enzymes and/or posttranslational modification(s). It was already shown that SOD can be inhibited by its own product—H_2_O_2_ [27, 29, 30]. On the other hand, increasing levels of H_2_O_2_ lead to GSH-Px and CAT activation by phosphorylation by the tyrosine kinase c-Abl/Arg complex by different kinetics [31, 32]. Moreover, CAT activity is under control of different phosphatases [33]; its inhibitory effects are suppressed by the excess of calcium [34]. Furthermore, it was shown that external Ca^2+^ addition induced significantly the generation of ROS and Ca^2+^ influx [34]; this can be one of the reasons way CAT initial activity levels are higher in Ca^2+^-stimulated uteri compared to spontaneously active uteri. However, the addition of ibogaine further deepened the state of oxidative stress, suggesting ibogaine-mediated oxidative input that led to a physiological response toward the establishment of ROS homeostasis.

## 5. Conclusions

Overall, in our experimental setting, ibogaine treatment altered the redox homeostasis and affected the contractile properties of the uterus. Changes in antioxidant enzyme activities point to a vast, concentration-dependent increase in cellular respiration and H_2_O_2_ level. The results show that ibogaine affects both spontaneously active and Ca^2+^-stimulated contractions. Low concentrations of ibogaine stimulated spontaneous contractions, which might be, at least in part, related to an increase in extracellular ATP. On the other hand, high ibogaine concentrations exhibited inhibiting effects on both spontaneously active and Ca^2+^-stimulated active uteri. This decrease in the contractile activity of isolated uteri is at least partially contributed by ibogaine-related alterations in redox homeostasis and changes in ROS equilibrium.
